# Air quality assessment based on R/S–SPA composite model: A case study of the Hebei province (China)

**DOI:** 10.3389/fpubh.2022.922125

**Published:** 2022-10-03

**Authors:** Qinfeng Xing, Ziwei Yang, Wanyan Yao

**Affiliations:** State Key Laboratory of Mining Response and Disaster Prevention and Control in Deep Coal Mines, Anhui University of Science and Technology, Huainan, China

**Keywords:** air quality, AQI analysis, R/S analysis, set pair analysis, Hebei province (China)

## Abstract

Air pollution has been the favorite subject of pollution prevention and control measures with the aim of sustainable development. Therefore, a composite model of rescaled range (R/S) and set pair analysis (SPA) was constructed to evaluate the air pollution situation based on the data from 2013 to 2020 in the Hebei province and then predict its air quality development trend. The results show that (1) the air pollution situation is severe, but the development trend is good and (2) the main pollutants are the core factors affecting air quality, and it is necessary to focus on intervention. The innovation of this paper lies on the combination of R/S and SPA to jointly predict the development trend of air quality in the Hebei province and ensure its scientific prediction. Meanwhile, this paper does not point out the continuation cycle of development state, which is the limitation of this paper. Finally, this study deepens the understanding of air quality evaluation, and the following countermeasures are formed: adherence to the problem- and goal-oriented approach to help the transformation of low-carbon development and enhancing the awareness of energy conservation and environmental protection to practice low-carbon lifestyle.

## Introduction

Air pollution is one of the outstanding problems for environmental security. In recent years, positive results have been achieved in the prevention and control of air pollution, and the phased targets and tasks for the prevention and control of air pollution have been gradually fulfilled. Total emissions of major air pollutants, total emissions per unit of gross domestic product (GDP), and fine particulate matter (PM_2.5_) concentrations continue to decline. However, the core contradiction of air pollution prevention and control runs deeper (1). The structural, root, and trend pressures of air pollution have not been fully resolved. These problems make it more difficult to achieve the goal of improving air quality. Furthermore, air pollution is affected by exhaust emissions from heavy industry, construction, and automobiles (2). Although the efforts devoted to structural adjustment have been made, the fundamental problems have not been effectively solved, and the improvement of air quality remains a long-term target.

As Hebei is an industrial province of China, the structural problem of air pollution in the Hebei province is increasingly prominent. Therefore, taking the Hebei province as a case study is of great value to the proposal of air pollution control plan, as well as for strengthening the stability of air quality to optimize its prevention and control measures. Furthermore, taking the prevention and control of air pollution as a research topic cannot only effectively improve air quality, but also lay the theoretical foundation for achieving the goal of “dual carbon.”

## Materials and methods

### Study area

**Figure 1:**
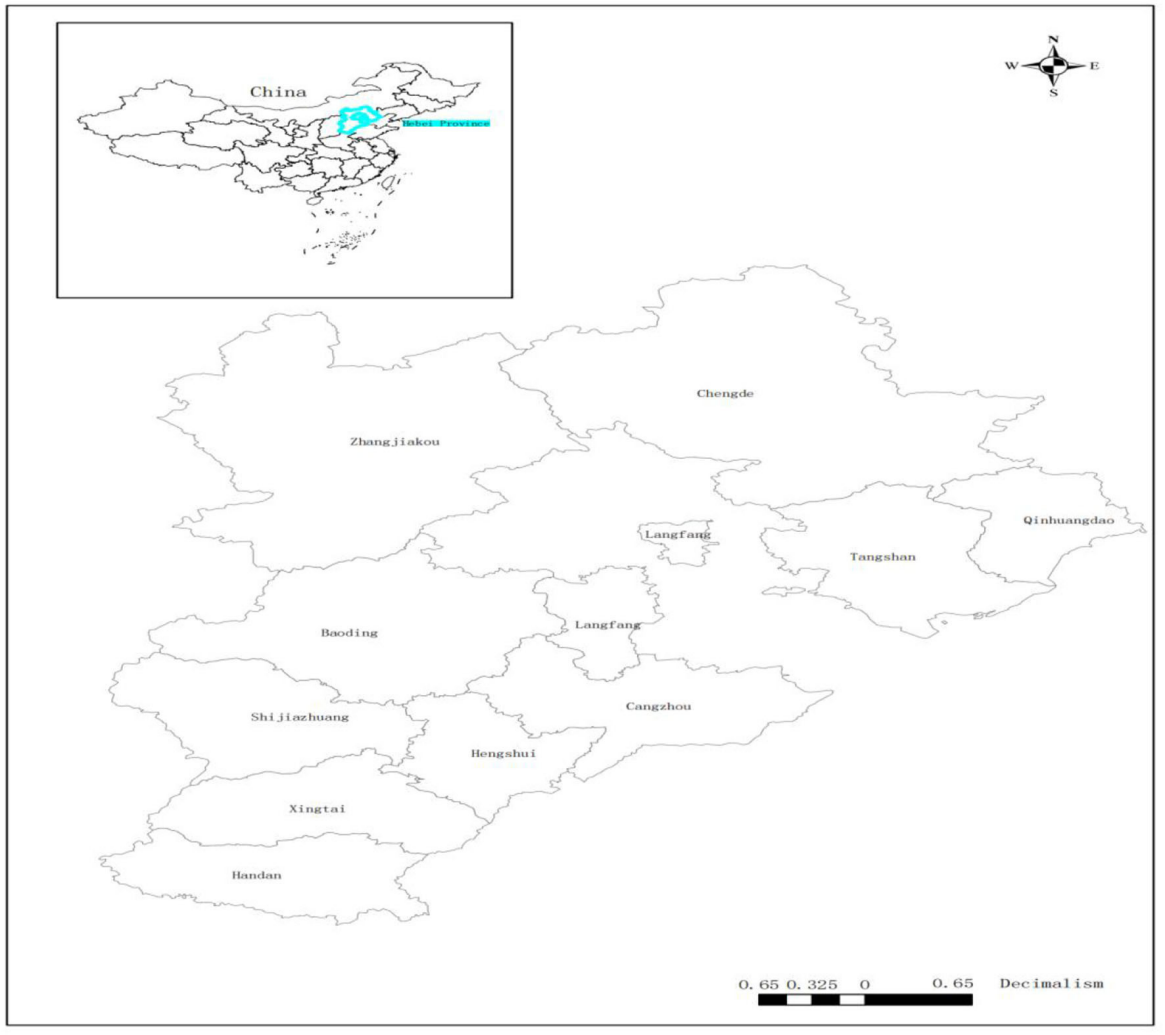
The geographical position of the Hebei province.

Hebei province is located in North China, north of Zhanghe River, east of Bohai Sea, west of Taihang Mountain, north of Yanshan, east longitude 113°27'−119°50', (shown in Figure 1) and north latitude 36°05'−42°40'. As an industrial province, it has heavy and chemical industries as an industrial structure, so the haze in the Hebei province becomes a direct embodiment of air pollution. Furthermore, the production activities of many enterprises are accompanied by high carbon emissions and high air pollution, so it is difficult to carry out environmental protection work to improve air quality. Additionally, emissions of sulfur dioxide (SO_2_), nitrogen dioxide (NO_2_), ozone (O_3_), and carbon monoxide (CO) are serious, and PM_2.5_ pollution has not been fundamentally controlled. Finding effective ways to address the prevention and control of carbon emissions to improve air quality has become a top priority. However, in the 5 years of the 13th 5-year plan (2016–2020), although problems such as industrial structure and energy structure have not been effectively solved, many measures have been gradually adopted by local governments and some entrepreneurs to improve air quality.

### Data sources

Air quality index (AQI) is a dimensionless index that quantifies air quality and represents the degree of air pollution in a region. As the concentration limits for the six pollutants evaluated by AQI are different, the individual air quality index (IAQI) will be calculated according to the different target concentration limits for each pollutant in the evaluation.

The data analysis in this paper is based on Hebei province Environmental Status and Quality Bulletin 2013–2020, GB 3095-2012 Environmental Air Quality Standard including Amendment No. 1 2018. Then, the follow-up research with IAQI of the corresponding area and the corresponding pollutant concentration index (Table 1) and the related information of AQI (Table 2) are calculated. Among them, IAQI is calculated from the measured concentrations of six pollutants as follows: PM_2.5_, inhalable particulate matter (PM_10_), SO_2_, NO_2_, O_3_, and CO. Meanwhile, PM_2.5_, PM_10_, SO_2_, NO_2_, and CO have a 24-h average basis, respectively, O_3_ has an 8-h moving average basis. The specific calculation process is presented as follows:

(1) The calculation formula of IAQI was expressed in Equation (1).

(1) $$\begin{array}{l} IAQI_{p}=\frac{IAQI_{Hi}-IAQI_{Lo}}{BP_{Hi}-BP_{Lo}}\left(C_{p}-BP_{Lo}\right)+IAQI_{Lo} \end{array}$$

where *IAQI*_*p*_—individual air quality index of the pollutant *P*.

(2) The AQI value was selected from the maximum IAQI value of the six pollutants in Equation (2).

(2) $$\begin{array}{l} AQI=\max\left\{IAQI_{1},IAQI_{2},\cdots IAQI_{p}\right\},p=1,2,\cdots \;,6 \end{array}$$

**Table 1:** Individual air quality index (IAQI) of the corresponding area and corresponding pollutant concentration index.

**IAQI**	**Pollutant concentration limit**					
	**SO_2_ (μg/m^3^)**	**NO_2_ (μg/m^3^)**	**PM_10_ (μg/m^3^)**	**CO (mg/m^3^)**	**O_3_ (μg/m^3^)**	**PM_2.5_ (μg/m^3^)**
0	0	0	0	0	0	0
50	50	40	50	2	100	35
100	150	80	150	4	160	75
150	475	180	250	14	215	115
200	800	280	350	24	265	150
300	1,600	565	420	36	800	250
400	2,100	750	500	48	-	350
500	2,620	940	600	60	-	500

IAQI will not be calculated if the average concentration of O_3_ is higher than 800 μg/m^3^ in 8 h. Furthermore, *C*_*p*_—mass concentration value of the pollutant P, *BP*_*Hi*_—The high value of the pollutant index concentration limit (it means the AQI of the corresponding area and corresponding pollutant concentration index), *BP*_*L*o_—the low value of the pollutant index concentration limit (it means the IAQI of the corresponding area and corresponding pollutant concentration index), *IAQI*_*Hi*_—IAQI corresponding to*BP*_*Hi*_ (it means the IAQI of the corresponding area and corresponding pollutant concentration index), *IAQI*_*Lo*_—IAQI corresponding to *BP*_*L*o_ (it means the IAQI of the corresponding area and corresponding pollutant concentration index), all are presented in Table 2.

**Table 2:** Related information of air quality index (AQI).

**AQI**	**AQI level**	**AQI categories and colors**
0–50	Level 1	Excellent	Green
51–100	Level 2	Good	Yellow
101–150	Level 3	Slightly pollution	Orange
151–200	Level 4	Moderate pollution	Red
201–300	Level 5	Severely pollution	Purple
>300	Level 6	Seriously pollution	Maroon

### Rescaled range model

The rescaled range (R/S) model was first proposed by Hurst (3). R/S is mainly used to analyze the terms persistence and anti-persistency to predict the future development trend of the Hurst index (4). Time series refers to the sequence formed by arranging the values of a certain statistical indicator at different times in a chronological order (5). R/S has been widely used in the study of a quantitative prediction system. Currently, R/S, spectrum analysis, periodic graph regression method, and correlation analysis method are mostly used when fractal characteristics of time series are analyzed (6–9). As R/S is more accurate than other methods to analyze the fractal structure characteristics of time series, it has an important reference value for air pollution. The main steps of R/S are given as follows (10):

(1) Suppose, at different times, *t*_1_, *t*_2_, ⋯, *t*_*n*_ the concentration of a pollutant in the atmosphere is *x*_1_, *x*_2_, ⋯, *x*_*n*_, then the time series of pollutant content is {*x*_*i*_, *n* ≥ *i* ≥ 1}, and the mean value of pollutant concentration in *k* (*k* ≥ 2) can be calculated by Equation (3).

(3) $$\begin{array}{l} \bar{x_{k}}=\frac{1}{n}\overset{n}{\underset{i=1}{\sum }}x_{i} \end{array}$$

(2) At time *t*_*j*_, the standard deviation (SD) of pollutant concentration from the mean value can be calculated by Equation (4).

(4) $$\begin{array}{l} S\left(k\right)=\sqrt{\frac{\overset{n}{\underset{i=1}{\sum }}{\left(x_{i}-x_{k}\right)}^{2}}{n}} \end{array}$$

(3) At time *t*_*j*_, the cumulative deviation of pollutant concentration from the mean value can be calculated by Equation (5).

(5) $$\begin{array}{l} x\left(t_{j},k\right)=\overset{j}{\underset{i=1}{\sum }}\left(x_{i}-\bar{x_{k}}\right) \end{array}$$

(4) The range of *x*(*t*_*j*_, *k*) can be calculated by Equation (6).

(6) $$\begin{array}{l} R\left(k\right)=\max x\left(t,k\right)-\min x\left(t,k\right),\\ \;t_{n}\geq t\geq t_{1},k=2,3,4,\cdots \end{array}$$

(5) The *R/S* of each subset and the (*R/S*)_*k*_ on the time span of *k* can be calculated by Equations (7, 8).

(7) $$R/S=\frac{R\left(k\right)}{S\left(k\right)}=\frac{\underset{t_{1}\leq t\leq t_{n}}{\max x\left(t,k\right)}-\underset{t_{1}\leq t\leq t_{n}}{\min x\left(t,k\right)}}{\sqrt{\frac{1}{n}\sum_{i=1}^{n}{\left(x_{i}-\bar{x_{k}}\right)}^{2}}},k=2,3,4,\cdots$$

(8) $$\begin{array}{l} {\left(R/S\right)}_{k}=\frac{1}{m}\overset{m}{\underset{j=1}{\sum }}{\left(\frac{R\left(k\right)}{S\left(k\right)}\right)}_{j} \end{array}$$

where *j* represents the type of air pollutants. Furthermore, six pollutants are studied in this paper, so *j* can be taken from 1 to 6.

(6) *k* starts from 2, the first five steps are repeated and gradually increase, then the sequence {(*R*/*S*)_*k*_} can be calculated.
(7) According to the definition of the Hurst index _*H*_, ordinary least squares (OLS) linear regression is performed by IBM SPSS23.0. Then, the Hurst index can be calculated by the Hurst index, *H*ε{0,1}. The specific analysis process is presented as follows: (1) when *H* > 0.5, all data meet a positive correlation or long memory, which means that the future trend of time series is consistent with the past trend. The closer the value is to 1, the longer the long memory. (2) When *H* = 0.5, it means that all data in the sequence are independent and unrelated, and the development trend of the preceding sequence has nothing to do with the change trend of the following sequence. (3) When *H* < 0.5, all or part of the data meet a negative correlation or anti-memory, which means that the trend in the future will be contrary to the past. Furthermore, the closer the value is to 0, the stronger the anti-memory (11). Moreover, the variation trend of the dispersion degree of the observed values can be obtained using the coefficient of variation (the ratio of SD to mean).
(8) *V*_*n*_ statistics expressed by Equation (9) can be used to estimate the length of the periods. It is first used to test stability and then to estimate the length of periods.

(9) $$\begin{array}{l} V_{n}=\frac{{\left(R,S\right)}_{n}}{\sqrt{n}}\; \end{array}$$

### Set pair potential analysis

Set pair analysis (SPA) was proposed by Zhao Keqin in 1989 and was applied in many fields (12). The central idea of SPA is to consider all factors as a system of interaction between certainty and uncertainty, and to describe these factors with identity, difference, and opposition (13). It is assumed that the set pair *A* is composed of six pollutants as the indicator set and the air quality assessment criteria set, and the set pair *A* is composed of *N* elements in both the indicator set and the assessment criteria set, where the number of elements with the same property (same state) in set *A* is *S*, the number of elements with opposite properties (opposite states) is *P*, and *F* = *N*−*S*−*P* is the number of elements with different properties (different states) in the set *A*. According to the relationship between them, the degree of relationship is expressed by Equation (10).


(10) $$\begin{array}{l} \mu =\frac{S}{N}+\frac{F}{N}i+\frac{P}{N}j=a+bi+cj \end{array}$$

where $\frac{S}{N}$, $\frac{F}{N}$, and $\frac{P}{N}$ represent the *A* identity degree, difference degree, and opposition degree of the set pair, respectively, denoted as *a*, *b*, and *c* in turn, and they are the difference degree coefficient and opposition degree coefficient *a*+*b*+*c* = 1, and *i* ∈ [−1, 1] *j* = −1, respectively. Furthermore, the potential analysis of air quality can be calculated using the set pair potential of the five-element relation number as follows: (1) when *c* ≠ 0, the set pair potential $shi\left(H\right)=\frac{a}{c}$ can be reached; (2) when $\frac{a}{c}>1$, the potential of two sets is the same, then the future development trend of the indicators is conducive to the improvement of the air quality level, and the development space of indicators should be further expanded; (3) when $\frac{a}{c}=1$, the potential of two sets is in equilibrium; (4) when $\frac{a}{c}<1$, the potential of two sets is the opposite, then the development trend of the indicators in the future is not conducive to the improvement of the air quality level, which needs to be paid more attention.

According to the above mentioned principle, the same–different–anti model can be constructed by Equation (11) (14, 15).


(11) $$\begin{array}{l} \begin{array}{c} \mu =WRE=\\ \left(\omega 1\;\omega 2\;\cdots \;\omega n-1\;\omega n\right)\left(\begin{array}{ccccc} u11 & u12 & u13 & u14 & u15\\ u21 & u22 & u23 & u24 & u25\\ u31 & u32 & u33 & u34 & u35\\ \cdots & \cdots & \cdots & \cdots & \cdots \\ un1 & un2 & un3 & un4 & un5 \end{array}\right)\left(\begin{array}{c} 1\\ \lambda \\ \gamma \\ \phi \\ \psi \end{array}\right)\\ =\overset{n}{\underset{j=1}{\sum }}\omega_{j}u_{j1}+\overset{n}{\underset{j=1}{\sum }}\omega_{j}u_{j2}\lambda +\overset{n}{\underset{j=1}{\sum }}\omega_{j}u_{j3}\gamma +\overset{n}{\underset{j=1}{\sum }}\omega_{j}u_{j4}\phi +\\ \;\overset{n}{\underset{j=1}{\sum }}\omega_{j}u_{j5}\psi =a+b_{1}\lambda +b_{2}\gamma +b_{3}\phi +c\psi \end{array} \end{array}$$

where $\left(\begin{array}{ccccc} \omega 1 & \omega 2 & \cdots & \omega n-1 & \omega n \end{array}\right)$ is the weight vector of six pollutants, {*u*_*ij*_} represents the evaluation matrix, and the evaluation matrix is obtained by using the formula $ujs{=}^{zjs}{/}_{z}$, *z* represents the number of evaluation experts, and *zjs* represents the number of people who think that *j* belongs to the grade *s*. Then, the degree of influence and probability of each indicator on air quality are analyzed. (1, λ, γ, ϕ, ψ)^*T*^ is the matrix of identical and different inverse coefficients. *a* and *c* are definite values, *b*_1_, *b*_2_, *b*_3_, *b*_2_, and *b*_3_ are uncertain values, *a*, *b*_1_, *b*_2_, *b*_3_, and *c*, respectively, represent the degree of unsafe, less safe, critical safe, relatively safe, and safe in air quality evaluation.

As the index is of the smallest type, the most optimal type in this paper, the detailed expression of the five-element single index connection number can be obtained by Equation (12).


(12) $$\mu =\{\begin{array}{l} \text{1}+\text{0}\lambda +0\gamma +0\phi +0\psi \left(x\leq s_{1}\right)\\ \frac{s_{\text{2}}-x}{s_{\text{2}}-s_{\text{1}}}+\frac{x-s_{\text{1}}}{s_{\text{2}}-s_{\text{1}}}\lambda +0\gamma +0\phi +0\psi \left(s_{\text{1}}x\leq s_{\text{2}}\right)\\ \text{0}+\frac{s_{3}-x}{s_{\text{3}}-s_{\text{2}}}\lambda +\frac{x-s_{\text{2}}}{s_{\text{3}}-s_{\text{2}}}\gamma +0\phi +0\psi \left(s_{\text{2}}x\leq s_{\text{3}}\right)\\ \text{0}+\text{0}\lambda +\frac{s_{\text{4}}-x}{s_{\text{4}}-s_{\text{3}}}\gamma +\frac{x-s_{\text{3}}}{s_{\text{4}}-s_{\text{3}}}\phi +0\psi \left(s_{\text{3}}x\leq s_{\text{4}}\right)\\ \text{0}+\text{0}\lambda +0\gamma +\frac{s_{\text{5}}-x}{s_{\text{5}}-s_{\text{4}}}\phi +\frac{x-s_{\text{4}}}{s_{\text{5}}-s_{\text{4}}}\psi \left(s_{\text{4}}x\leq s_{\text{5}}\right)\\ \text{0}+\text{0}\lambda +0\gamma +0\phi +1\psi \left(xs_{5}\right) \end{array}$$

where *x* is the evaluation index value, and *s*_1_, *s*_2_, *s*_3_, *s*_4_, and *s*_5_ are the critical values of index concentration grade, respectively.

The single index linkage degree can be obtained from Equation (12), and then in combination with Equation (11), the comprehensive index linkage degree is obtained. To determine the corresponding relationship between the comprehensive linkage degree and the judgment interval of risk grade, the five-value equal proportion principle was introduced to divide the interval [−1, 1] into equal parts (Table 3). The corresponding values of λ, γ, and ϕ were obtained by the principle of equipartition, and ψ = −1, so as to obtain the comprehensive correlation degree of air quality in the Hebei province.

**Table 3:** Air quality safety level judgment interval.

**Interval**	**[−1, −0.6]**	**[−0.6, −0.2]**	**[−0.2, 0.2]**	**[0.2, 0.6]**	**[0.6, 1]**
Grade	I	II	III	IV	V
Level	Unsafe	Less safe	Critical safe	Relatively safe	Safe

And, the values of λ, γ, and ϕ are obtained from Equation (13).


(13) $$\begin{array}{l} i_{l}=\frac{5-\left(2l+1\right)}{5-1},\left(l=1,2,3\right) \end{array}$$

where *i*_1_, *i*_2_, and *i*_3_ correspond to λ, γ, and ϕ, respectively.

In Table 3, the comprehensive correlation degree of air quality safety in the Hebei province can be presented. Furthermore, to predict the trend, the five-element partial correlation number and the correlation degree are given as follows:

First-order partial connection number can be obtained from Equation (14).

(1) First-order partial connection number can be obtained from Equation (14).

(14) $$\begin{array}{l} \partial u=\partial a+\lambda \partial b_{1}+\gamma \partial b_{2}+\phi \partial b_{3} \end{array}$$

where $\partial a=\frac{a}{a+b_{1}}$, $\partial b_{1}=\frac{b_{1}}{b_{1}+b_{2}}$ $\partial b_{2}=\frac{b_{2}}{b_{2}+b_{3}}$, and $\partial b_{3}=\frac{b_{3}}{b_{3}+c}$

(2) Second-order partial relation number can be obtained from Equation (15).

(15) $$\begin{array}{l} \partial^{2}u=\partial^{2}a+\lambda \partial^{2}b_{1}+\gamma \partial^{2}b_{2} \end{array}$$

where $\partial^{2}a=\frac{\partial a}{\partial a+\partial b_{1}}$, $\partial^{2}b_{1}=\frac{\partial b_{1}}{\partial b_{1}+\partial b_{2}}$, and $\partial^{2}b_{2}=\frac{\partial b_{2}}{\partial b_{2}+\partial b_{3}}$

(3) Third-order partial correlation number can be obtained from Equation (16).

(16) $$\begin{array}{l} \partial^{3}u=\partial^{3}a+\lambda \partial^{3}b_{1} \end{array}$$

where $\partial^{3}a=\frac{\partial^{2}a}{\partial^{2}a+\partial^{2}b_{1}}$ and $\partial^{3}b_{1}=\frac{\partial^{2}b_{1}}{\partial^{2}b_{1}+\partial^{2}b_{2}}$

(4) Fourth-order partial relation number can be obtained from Equation (17).

(17) $$\begin{array}{l} \partial^{4}u=\partial^{4}a \end{array}$$

where $\partial^{4}a=\frac{\partial^{3}a}{\partial^{3}a+\partial^{3}b_{1}}$

## Results

### Status analysis

#### Cp analysis

**Figure 2:**
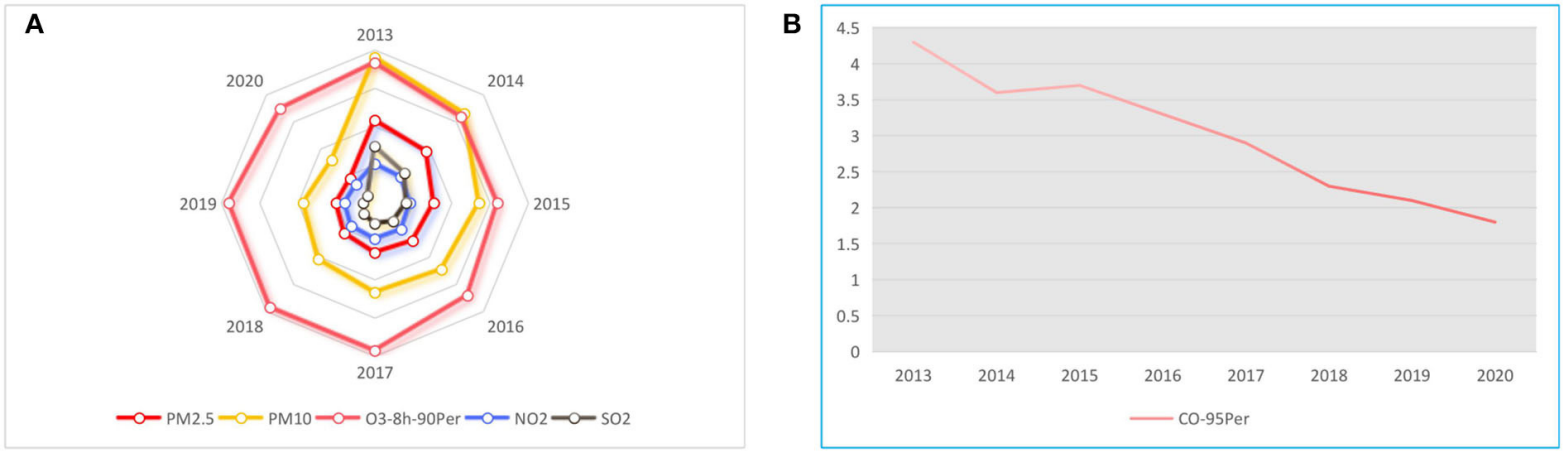
Annual mean change trend of Cp of six pollutants in the Hebei province (2013–2020). **(A)** Annual mean value of Cp. **(B)** Annual mean value of Cp. Cp is the mass concentration value of the pollutant P.

Through data collection, concentrations of various air pollutants in the Hebei province from 2013 to 2020 are presented in Figure 2A, and the corresponding trend chart of CO is presented in Figure 2B.

In Figure 2, the unit of PM_2.5_, PM_10_, O_3_, NO_2_, and SO_2_ is μg/m^3^ and the unit of CO is mg/m^3^. Then, the figures are presented separately. Furthermore, the six indicators involved in the monitoring of the concentration of major air pollutants in the Hebei province show a decreasing trend of varying degrees in 2013–2020. Among them, the fluctuation range of PM_2.5_, PM_10_, SO_2_, and CO is large, but the fluctuation range of O_3_ and NO_2_ is small. The values of PM_2.5_, PM_10_, and SO_2_ have been declining, and the three indicators including CO, O_3_, and NO_2_ show an upward trend in 2015, 2015–2019, and 2016, respectively, and then decline.

#### IAQI and AQI analysis

According to Equations (1, 2), the IAQI and AQI of major air pollutants in the Hebei province from 2013 to 2020 are presented in Table 4.

**Table 4:** IAQI and AQI values in the Hebei province (2013–2020).

	**IAQI**						
**Year**	**PM_2.5_**	**PM_10_**	**O_3_-8h-90Per**	**NO_2_**	**CO-95Per**	**SO_2_**	**AQI**
2013	141.25	120.00	120.91	77.50	101.50	74.00	141.25
2014	125.00	107.50	148.33	70.00	130.00	55.00	148.33
2015	102.50	136.00	150.00	65.00	135.00	41.00	150.00
2016	137.50	123.00	110.00	72.50	115.00	34.00	137.50
2017	125.00	117.00	130.00	67.50	95.00	27.00	130.00
2018	102.50	104.00	130.00	57.50	65.00	20.00	130.00
2019	88.00	93.00	127.27	48.75	55.00	15.00	127.27
2020	74.50	79.00	112.73	42.50	45.00	13.00	112.73

O_3_-8h-90Per represents the maximum 8-h mean 90th percentile concentration of O_3_ daily. CO-95Per represents the 95th percentile concentration of CO.

In Table 4, it was found that, in the IAQI, SO_2_ decreased year-by-year. Meanwhile, the other five pollutants such as PM_2.5_, PM_10_, O_3_-8h-90Per, NO_2_, and CO-95Per increased and later decreased. Furthermore, AQI also rose and later fell. It means that the air quality has been slowly getting better.

### Predictive analysis

#### Analysis of coefficient of variation

**Figure 3:**
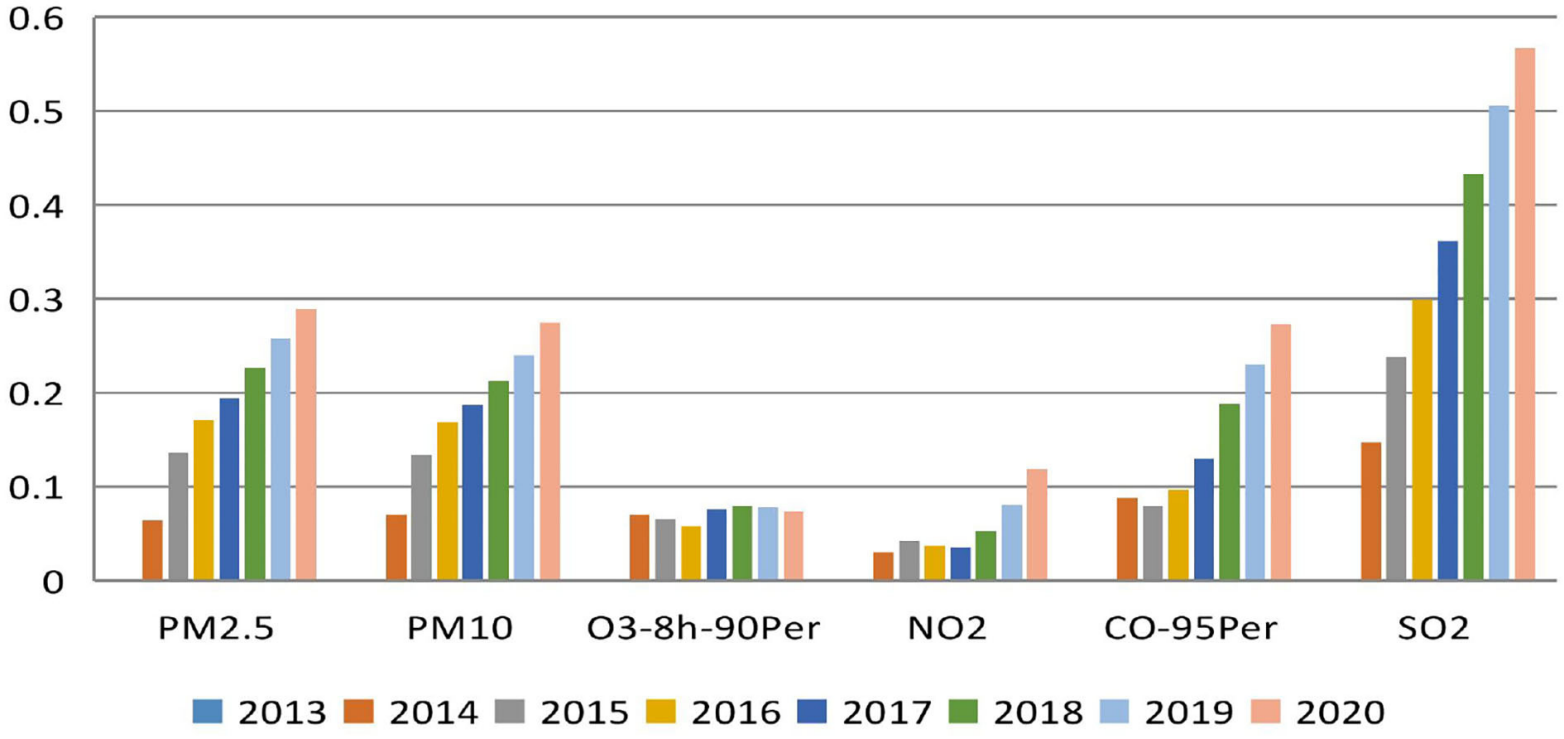
Variation coefficient of the air quality index (AQI) indicator in the Hebei province (2013–2020).

In combination with the annual mean and SD of pollutant concentrations obtained from Equations (3, 4) and the calculation principle of variation coefficient (mean divided by SD), the variation coefficient of pollutants in the Hebei province from 2013 to 2020 is presented in Figure 3.

In Figure 3, the main use of variation coefficient is that it can directly observe the size of the fluctuation range of main air pollutants, which is convenient for the follow-up targeted treatment. Furthermore, air quality is affected by six major pollutants, and variation coefficients, such as PM_2.5_, PM_10_, and SO_2_, show an increasing trend. Among them, the dispersion degree of SO_2_ is the largest, indicating that its value fluctuates violently; the variation coefficients, such as CO, O_3_, and NO_2_, reach the minimum in 2015, 2016, and 2017, respectively. Then, they increase year-by-year. Except O_3_, the other two indicators show significant increases in 2018–2020, indicating that their values fluctuate greatly in recent years. In combination with the annual mean value of *C*_*p*_ initially, the maximum variation ranges from high to low as follows: SO_2_, PM_2.5_, CO, PM_10_, NO_2_, and O_3_ indicate that effective results have been achieved in the collaborative control of air pollutants in recent years, and the prevention and control of air pollution has helped to continuously improve air quality.

#### R/S analysis

In combination with Equations (3–9), the time series of IAQI of major air pollutants in the Hebei province from 2014 to 2020 and the time series value of AQI are presented as in Table 5.

**Table 5:** AQI analysis in the Hebei province.

		**ln (R/S)**_**k, j**_						
**Year**	**Lnk**	**PM_2.5_**	**PM_10_**	**O_3_-8h-90Per**	**NO_2_**	**CO-95Per**	**SO_2_**	**ln (R/S)_k_**
2014	0.69	0	0	0	0	0	0	0
2015	1.10	0.25	0.23	0.35	0.26	0.34	0.25	0.28
2016	1.39	0.63	0.61	0.59	0.51	0.46	0.57	0.56
2017	1.61	0.83	0.82	0.81	0.49	0.69	0.78	0.74
2018	1.79	0.95	0.95	1.03	0.63	0.90	0.92	0.90
2019	1.95	1.08	1.08	1.18	1.02	1.11	1.07	1.09
2020	2.08	1.20	1.19	1.20	1.21	1.26	1.20	1.21

**Figure 4:**
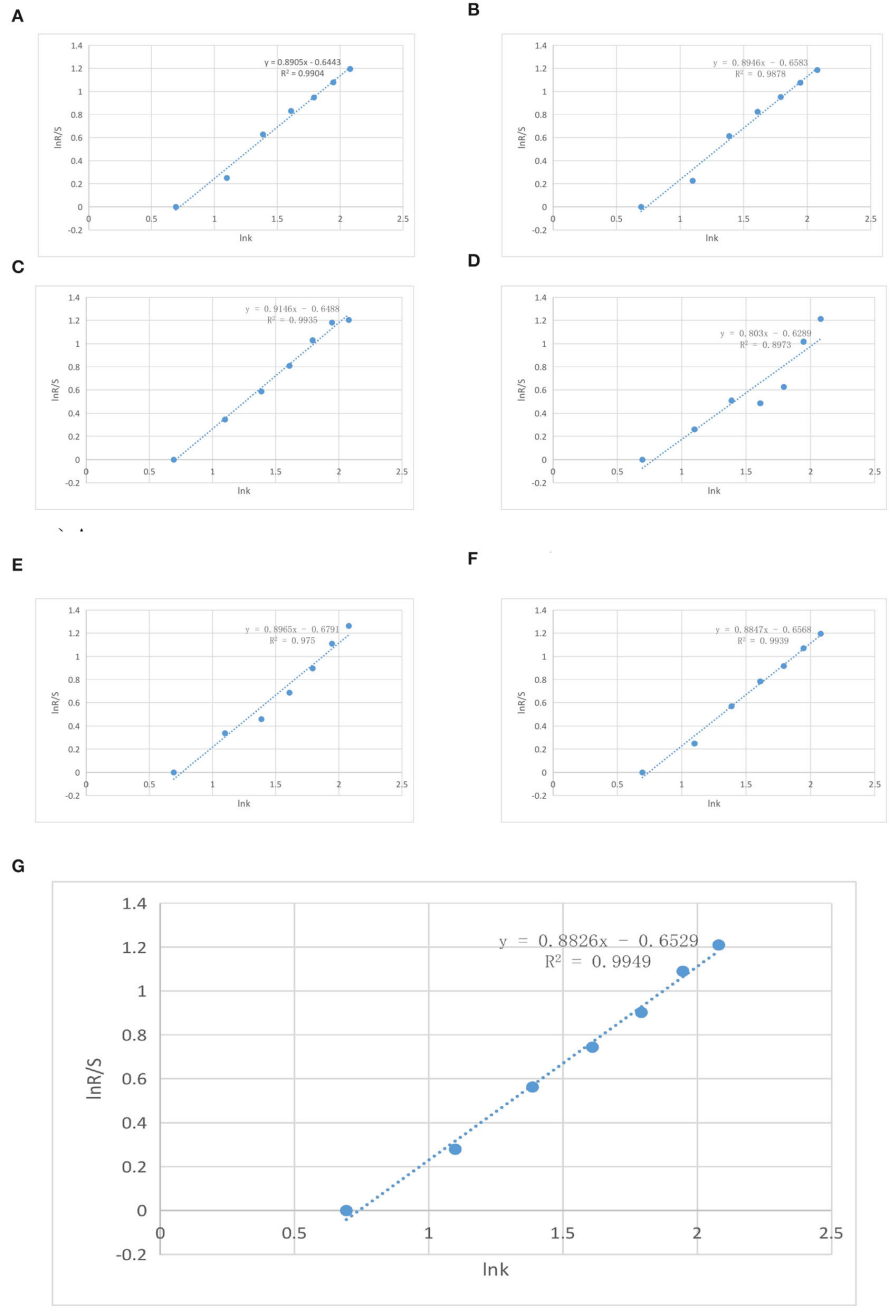
Rescaled range (R/S) analysis results of AQI in the Hebei province from 2013 to 2020. **(A)** Analysis result of PM_2.5_. **(B)** Analysis result of PM_10_. **(C)** Analysis result of O_3_. **(D)** Analysis result of NO_2_. **(E)** Analysis result of CO. **(F)** Analysis result of SO_2_. **(G)** The results of comprehensive analysis.

In Table 5, 2013 is the 1st year of the study, *k* represents the time span from 2013, for example, 2014 is the 2nd year of the study, *k* = 2, 2015 is the 3rd year of the study, *k* = 3, and so on. Furthermore, the logarithmic coordinate values of major air pollutants in the Hebei province from 2014 to 2020 are fitted by IBM SPSS23.0 *via* the least squares method in Figure 4.

As shown in Figure 4, the corresponding Hurst index and a significant indicator *R*^2^ are presented in Table 6.

**Table 6:** Hurst indicator and *R*^2^ of main air pollutants in the Hebei province.

**Year**	**PM_2.5_**	**PM_10_**	**O_3_-8h-90Per**	**NO_2_**	**CO-95Per**	**SO_2_**	**AQI**
Hurst index	0.8905	0.8946	0.9146	0.803	0.8965	0.8847	0.8826
*R* ^2^	0.9904	0.9878	0.9935	0.8973	0.975	0.9939	0.9949

**Figure 5:**
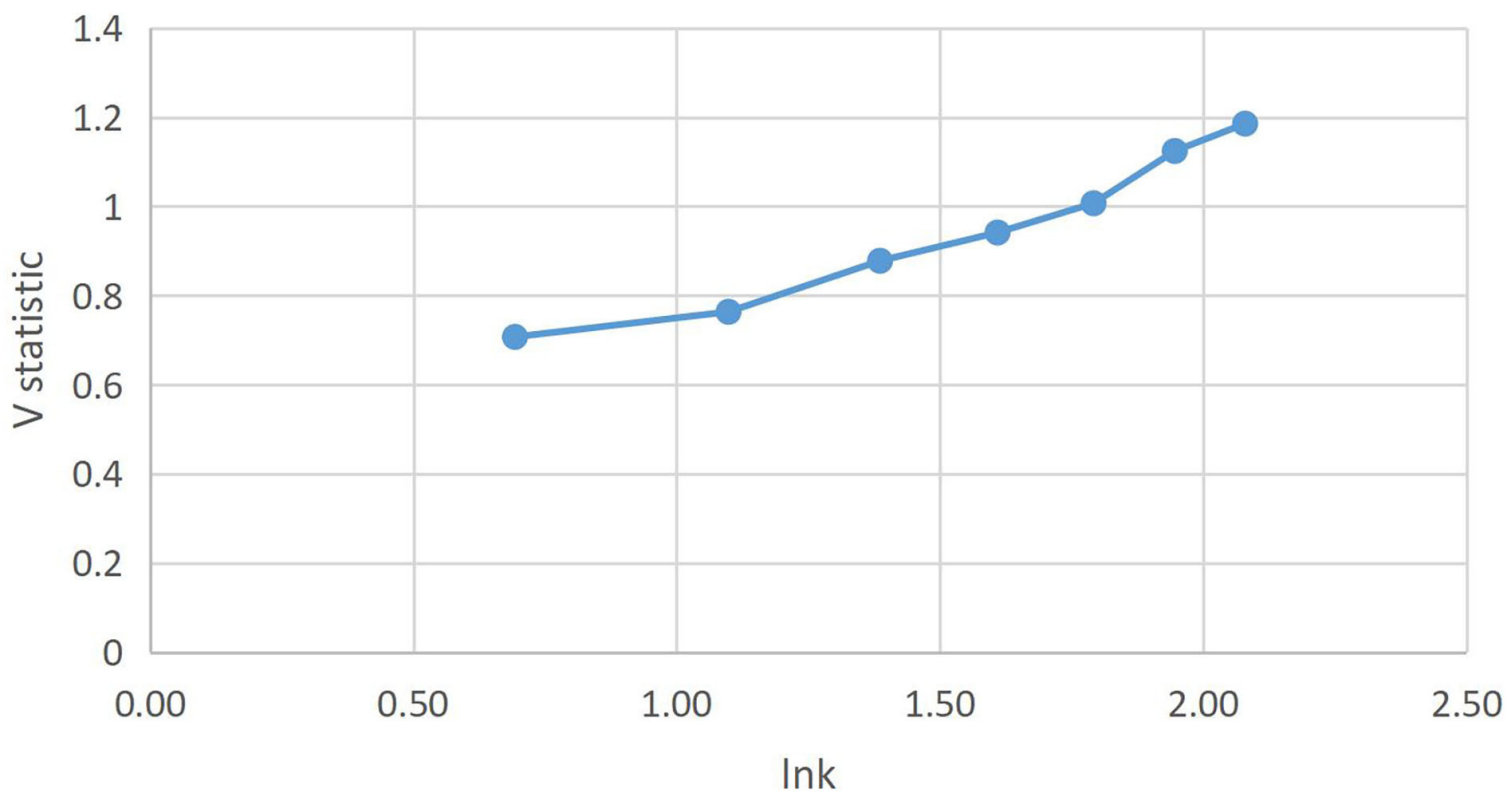
AQI *V* statistics of the Hebei province.

Finally, According to Tables 5, 6 and Figure 4, these results can be presented as follows:

1) It can be found that the fitting effect seems to be ideal when using the raw data from 2013 to 2020. For example, in combination with Figure 4 and Table 6, all seven fitting curves have a significant regression effect, which is >0.9. The significance coefficient for NO_2_ is 0.8973, which is very small and negligible. Furthermore, all seven *p*-values obtained by IBM SPSS23.0 are <0.05, which further verify that the regression equation has a high degree of synergy with reality and can predict air quality in the future.
2) According to the inherent meaning of the Hurst index, it can be found that the *H*-value of all major air pollutants is between 0.5 and 1. In combination with the practical principle of R/S, the time series involved has persistence. Furthermore, the trend of future AQI is fitted and predicted using Equation (9), and the results are presented in Figure 5.

In Figure 5, it can be seen that the length of acyclic cycle on air quality in the Hebei province has not been obtained, which may be due to the size of the sample data. However, based on the current development trend, the Hurst index is 0.9949 > 0.5 and the sequence has a valid continuous state. Furthermore, Vn vs. Lnn slopes upward, so air quality will continue to improve.

### Validation analysis

Through R/S analysis, the future development trend of air quality in the Hebei province is predicted and the abovementioned conclusions are obtained. However, its accuracy still needs to be verified. Therefore, this study evaluated its rationality by combining set-pair potential analysis. Furthermore, by combining Equations (10–17), the five-element correlation number of air quality of major air pollutants is presented in Table 7.

**Table 7:** Five-element correlation number of a sub-indicator in the Hebei province (2013–2020).

	**Five—member relation number**							
	**2013**	**2014**	**2015**	**2016**	**2017**	**2018**	**2019**	**2020**
PM_2.5_	0.175λ + 0.825γ	0.5λ + 0.5γ	0.95λ + 0.05γ	0.125 + 0.875λ	0.25 + 0.75λ	0.25 + 0.75λ	0.25 + 0.75λ	0.25 + 0.75λ
PM_10_	0.6λ + 0.4γ	0.85λ + 0.15γ	0.14 + 0.86λ	0.27 + 0.73λ	0.33 + 0.67λ	0.46 + 0.54λ	0.57 + 0.43λ	0.71 + 0.29λ
O_3_-8h-90Per	0.582λ + 0.418γ	0.017 + 0.983λ	Λ	0.8λ + 0.2γ	0.4λ + 0.6γ	0.4λ + 0.6γ	0.455λ + 0.545γ	0.745λ + 0.255γ
NO_2_	0.725 + 0.275λ	0.8 + 0.2λ	0.85 + 0.15λ	0.775 + 0.225λ	0.825 + 0.175λ	0.925 + 0.075λ	1	1
CO-95Per	0.97λ + 0.03γ	0.2 + 0.8λ	0.15 + 0.85λ	0.35 + 0.65λ	0.55 + 0.45λ	0.85 + 0.15λ	0.95 + 0.05λ	1
SO_2_	0.76 + 0.24λ	0.95 + 0.05λ	1	1	1	1	1	1

λ, γ, are the corresponding difference coefficient.

Later, according to Table 7, the corresponding partial correlation number is presented in Table 8.

**Table 8:** The number of second- and fourth-order partial connections in the Hebei province (2013–2020).

**Year**	**Second order partial connection number**	**Trend**	**Fourth order partial relation number**	**Trend**
2013	0.2475 + 0.474λ + 0.325γ	Decrease	0.485	Improve
2014	0.328 + 0.564λ + 0.108γ	Improve	0.610	Improve
2015	0.357 + 0.627λ + 0.008γ	Improve	0.671	Improve
2016	0.42 + 0.545λ + 0.033γ	Improve	0.693	Improve
2017	0.4925 + 0.4075λ + 0.1γ	Improve	0.696	Improve
2018	0.581 + 0.319λ + 0.1γ	Improve	0.741	Improve
2019	0.628 + 0.281λ + 0.091γ	Improve	0.769	Improve
2020	0.66 + 0.2975λ + 0.0425γ	Improve	0.809	Improve

λ, γ, are the corresponding difference coefficient.

In Table 8, the six major air pollutants involved in this study have a serious impact on air quality, and their weights are evenly distributed in this discussion.

Finally, according to Tables 7, 8, the results in the Hebei province during 2013–2020 were obtained as follows:

(1) The development trend of PM_2.5_, PM_10_, and CO is in the state of equilibrium ($\frac{a}{c}=1$) with the improvement of air quality at first, then in the state of homogeneity ($\frac{a}{c}>1$); the development trend of SO_2_ and NO_2_ is still in the state of homogeneity ($\frac{a}{c}>1$) with the improvement of air quality; the development trend of O_3_ is in the change process of equilibrium–homogeneity–equilibrium ($\frac{a}{c}=1\to \frac{a}{c}>1\to \frac{a}{c}=1$) with the improvement of air quality.
(2) The number of second-order partial connections in the Hebei province shows a decreasing trend in 2013, but an increasing trend from 2014 to 2020. The number of fourth-order partial connections shows an increasing trend from 2013 to 2020. This shows that air quality is in good trend, and the R/S analysis results are consistent, which prove the study of scientific rationality. Meanwhile, a lot of progress in the Hebei province has been made in dealing with the problems of air pollution, such as a significant reduction in the number of days with heavy air pollution and continued decline in peak PM_2.5_ levels. Furthermore, to further improve air quality, detailed action plans need to be formulated and strictly implemented.

## Discussion

Through the abovementioned analysis, the following discussion can be obtained:

(1) Air pollution situation is severe, but the development trend is good. According to the abovementioned data analysis results and the comparison in Table 2, it is found that the AQI in the Hebei province from 2013 to 2020 is in the stage of less pollution. According to the variation trend of coefficient of variation (Figure 2), the concentration of major pollutants is in a declining state. Meanwhile, the R/S analysis results and Hurst index also show that the concentration of major pollutants and the AQI will have a good prospect in the future.

Furthermore, in combination with the current situation and forecast results of air quality in the Hebei province, the total emission of major pollutants continues to decline, the emission reduction of SO_2_, NO_2_, and CO has achieved the binding targets of the 13th 5-Year Plan in 2019. The average concentration of PM_2.5_, PM_10_, and SO_2_ decreased significantly year-on-year, with PM_2.5_ decreased by 5.8% year-on-year, PM_10_ decreased by 6.1% year-on-year, and SO_2_ decreased by 21.1% year-on-year. However, the future air quality work should not be relaxed, and it is necessary to strengthen the prevention and control of air pollution (16), such as reducing the emission of air pollutants from industrial enterprises, strengthening the coordinated management of ozone air pollution in the Hebei province, and strengthening the in-depth management of mineral dust.

Additionally, efforts should be made to adjust the energy mix, cut production capacity in heavily polluting industries, and upgrade polluting production technologies to help air quality continue to improve. Under the guidance of the “dual carbon” target, the steady improvement of air quality will surely be realized.

(2) The main pollutants are the core factors affecting air quality, and it is necessary to focus on intervention. Through the AQI analysis results of major pollutants, it is found that IAQI is in the process of improvement to different degrees, and the change of coefficient of variation more deeply shows the change state of the major pollutants. Through the analysis of the abovementioned situation, it is found that the concentration of the main pollutants has different degrees of reduction, and plays an important role in air quality. Combined with the analysis results of R/S and SPA, it is found that the major pollutants will have a positive trend in the future. However, due to the lack of sample data, this paper does not get the continuous cycle of a positive trend, which is the limitation of this study.

Furthermore, according to the academic point (17), the factors influencing air quality containing waste gas, automobile exhaust, dust, fog, etc. are the objects of research in this paper. For example, the emission of PM_10_ is a key to improve air quality, so it is necessary to strengthen the reduction of emissions from relevant pollution sources. Meanwhile, SO_2_, NO_2_, and other sources of air pollution have different degrees of impact on air pollution due to different natural resources, geographical locations, socio-economic factors in different cities, and it is equally important to control natural and man-made sources of these pollutants (18).

Additionally, ecological environment and meteorological departments should implement the normal working mechanism in the analysis of the types of pollutant, the scope of pollution, and the change trend of pollution in heavy pollution weather. For example, it is important to take targeted, scientific, and law-based pollution control measures to speed up progress in air quality improvement.

## Conclusion

Based on the discussion of the abovementioned data analysis and results, two conclusions are obtained to improve the air quality in the Hebei province as follows:

(1) Adherence to the problem- and goal-oriented approach to help the transformation of low-carbon development.

“Imbalance” is the biggest obstacle in the prevention and control of air pollution in the Hebei province. To promote industrial construction and economic growth, the resource consumption capacity in the Hebei province is at the forefront. The imbalance among energy, industry, transportation, land use structure, and other aspects is the main driving force of air pollution. Facing the “bottleneck period” of overcoming difficulties (19), weak points should be recognized, and the targeted local policies should be adopted to deal with the problems of air pollution. For example, it is necessary to improve an existing way of coal utilization, actively develop advanced ways and technologies to ensure that coal can be fully burned and utilized, and reduce the pollution caused by coal utilization as far as possible. Furthermore, facing the problems of energy utilization, the relevant personnel also need to increase the use of clean energies, such as solar energy, wind energy, and geothermal energy. Moreover, to improve the utilization rate of clean energy on the whole, government departments should increase the publicity of clean energy and introduce a number of preferential policies to make clean energy deeply involved in people's daily production and life. Meanwhile, air pollution from industrial production should be focused. For example, the modernization of industrial production as the main target should be focused to adjust the industrial structure to crack down on high-pollution and high-energy-consumption industries. Additionally, to improve air quality, the “dual carbon” strategy should be implemented to strengthen air pollution control (20). And then, various emission reduction measures can be guaranteed in place to maximize emission reduction benefits.

(2) Enhancing the awareness of energy conservation and environmental protection to practice low-carbon lifestyle.

Combined with the above analysis results, air quality in the Hebei province can be further improved. For example, practicing low-carbon lifestyle is necessary for residents. This is because that a great loss to the health of residents has been caused by air pollution (21). Furthermore, due to the geographical location, some northern cities used to burn heaters in winter, which is one of the important reasons for aggravating air pollution. Therefore, residents' unhealthy living habits will also contribute to the aggravation of air pollution. Moreover, in cities with high population density, the discharge of domestic wastewater, industrial and traffic pollutants, and household garbage will also cause air pollution. Therefore, the knowledge of environmental protection should be popularized. And then, residents can establish the awareness of air quality protection. This is because residents are both victims of air pollution and beneficiaries of air pollution control, which depends on cooperation between relevant environmental protection agencies and residents. If urban residents can support the prevention and control of air pollution, air quality in China will achieve fundamental progress.

## Data availability statement

The original contributions presented in the study are included in the article/supplementary material, further inquiries can be directed to the corresponding author/s.

## Author contributions

QX: conceptualization, literature search, literature analyze, and writing review. ZY and WY: conceptualization and writing review and editing. All authors agreed with the content and all gave explicit consent to submit the manuscript.

## Funding

This work was supported by the following program: Anhui Provincial Philosophy and Social Science funding project: research on the index system of Rural revitalization of Revolutionary Fellow villagers in Dabie mountains (No. AHSKQ2021D19).

## Conflict of interest

The authors declare that the research was conducted in the absence of any commercial or financial relationships that could be construed as a potential conflict of interest.

## Publisher's note

All claims expressed in this article are solely those of the authors and do not necessarily represent those of their affiliated organizations, or those of the publisher, the editors and the reviewers. Any product that may be evaluated in this article, or claim that may be made by its manufacturer, is not guaranteed or endorsed by the publisher.

## References

[1] Wang X Ni YL Feng XY. Research on “dual carbon” target and strategy of central enterprises. Enterprise Manag. (2021) 483:50–7. 10.3969/j.issn.1003-2320.2021.11.022

[2] Wang H Li H Wang H Wang SL Zhang WJ. Study on the difference between pollution reduction and carbon reduction and regional economic development level in China. Chinese J Environ Eng Technol. (2021) 2021:1–15. 10.12153/j.issn.1674-991X.20210268

[3] Hurst HE. Long-term storage capacity of reservoirs. Trans Am Soc Civil Eng. (1951) 116:6518. 10.1061/TACEAT.0006518

[4] Wang XJ Jiang RG Xie JC Wang N Li XC. Study on runoff variation characteristics of weihe River mainstream based on fractal and R/S analysis. J Water Resour Water Transport Eng. (2019) 173:102–8. 10.16198/j.cnki.1009-640X.2019.01.013

[5] Xu ZF Zou JH Li C Yuan QY. Hurst index analysis of wind speed time series based on R/S analysis method. J Power Eng. (2019) 39:585–604. 10.3969/j.issn.1674-7607.2019.07.010

[6] Gong YZ Zhou LT Liu ZK. Precipitation warning index threshold model based on rainfall data. Water Resour Protect. (2021) 37:13–26. 10.3880/j.issn.1004-6933.2021.06.003

[7] Xiao Q Xuan YY. Construction and application of FCI in China based on mixing Dynamic Factor Model. Math Statist Manag. (2022) 41:333–48. 10.13860/j.cnki.sltj.20211207-001

[8] Wei MM Liu ZF Li CL Sun J. Rough estimation of carrier frequency offset of high dynamic signal based on interpolation and periodic graph method. Appl Res Comput. (2021) 39:548–56. 10.19734/j.issn.1001-3695.2021.07.0303

[9] Xu H Liu Q. NDVI change and its relationship with climatic factors in Yunnan Province from 2001 to 2019. Res Soil Water Conserv. (2022) 29:162–8. 10.13869/j.cnki.rswc.2022.01.018

[10] Chen ZJ Li J Fei Y Yu YH. Characteristics and trends of air pollution in Zhangjiagang City from 2011 to 2020. Sichuan Environ. (2021) 40:61–5. 10.14034/j.cnki.schj.2021.05.010

[11] Chen F Zhang ZQ Li F Sun KQ. Runoff prediction of Dage Hydrological Station in Miyun Reservoir based on EEMD decomposition and BOA algorithm optimization neural Network. J Northwest Forest College. (2021) 36:188–94. 10.3969/j.issn.1001-7461.2021.06.27

[12] Liang JW Su Q Qian HX He CF Shi LC. Safety risk assessment of slope support engineering based on game theory and set pair analysis. Railway Standard Design. (2021) 2021:1–9. 10.13238/j.issn.1004-2954.202103020001

[13] Jiang FC Zhou CC Ma QD. Risk assessment of pilotage in inland River based on combined weight set pair analysis model. J Saf Environ. (2021) 21:990–6. 10.13637/j.issn.1009-6094.2019.1668

[14] Li H Tian YQ Zhong XR. Study on the sameness and difference and inverse model of comprehensive evaluation of occupational hazards in coal mine workplace. J Saf Environ. (2012) 12:216–9. 10.3969/j.issn.1009-6094.2012.03.052

[15] Yang J Lu YB Jiang WJ Li N Wang DF Shi YL. Quality assessment of gaseous pollutants (CO, SO_2_, NO) in ambient air. China Environ Monitor. (2021) 37:178–85. 10.19316/j.issn.1002-6002.2021.06.19

[16] Su J Huang GQ He T Bai L. Analysis on the cascade propagation model of ecosystem vulnerability affected by mineral dust. Operat Res Manag Sci. (2021) 30:183–9. 10.12005/orms.2021.0000

[17] Shi KH Ding RJ Wu LF Zheng YF. Construction of a new grey system multivariate model for air quality prediction: a case study of Shijiazhuang City. J Syst Sci. (2022) 31:75–81.

[18] Zhang Y Li ZQ Zhao SH Zhang XY Lin JT Qin K. A review on collaborative observation of atmospheric pollutant gases and atmospheric particulate matter by satellite. J Remote Sens. (2022) 26:873–96. 10.11834/jrs.20211392

[19] Lu YF Wang J Yu CH Ma Y Gao HW. Spatial-temporal variation characteristics and influencing factors of fog and haze days in Qingdao city. J Ocean Univ China. (2021) 51:34–45. 10.16441/j.carol

[20] Fu JH Zhou FZ. Chinese provincial air quality measurement and influencing factors analysis. J Urban Probl. (2020) 12:20–7. 10.13239/j.b33124325

[21] Liu QQ Dang YX Zhang WZ Wei LY. Effects of PM2.5 pollution on subjective well-being and willingness to pay in Chinese cities. Geograph Sci. (2021) 9:2096–106. 10.13249/j.carol

